# Hsa_circ_0004872 alleviates meningioma progression by sponging miR-190a-3p/PTEN signaling

**DOI:** 10.1186/s12885-024-12084-1

**Published:** 2024-03-18

**Authors:** Yongkai Huang, Zhihui Wu, Zewei Peng, Anmin Liu, Wen Yuan, Deqing Han, Junmin Peng

**Affiliations:** 1 Neurosurgery Department, Zhuzhou Hospital Affiliated to Xiangya Medical College, Central South University, 412000 Zhuzhou, Hunan Province China; 2 Surgery Department, Zhuzhou Hospital Affiliated to Xiangya Medical College, Central South University, 412000 Zhuzhou, Hunan Province China; 3 Emergency Department, Zhuzhou Hospital Affiliated to Xiangya Medical College, Central South University, 412000 Zhuzhou, Hunan Province China; 4 Department of Anesthesiology, Zhuzhou Hospital Affiliated to Xiangya Medical College, Central South University, 412000 Zhuzhou, Hunan Province China

**Keywords:** Meningioma, hsa_circ_0004872, miR-190a-3p, PTEN/PI3K/AKT pathway

## Abstract

**Background:**

Meningioma, the most prevalent intracranial tumor, possesses a significant propensity for malignant transformation. Circular RNAs (circ-RNAs), a class of non-coding RNAs, have emerged as crucial players in tumorigenesis. This study explores the functional relevance of hsa_circ_0004872, a specific circ-RNA, in the context of meningioma.

**Methods:**

Molecular structure and stability of hsa_circ_0004872 were elucidated through PCR identification. Meningioma cell proliferation and apoptosis were assessed using the CCK-8 assay and flow cytometry, respectively. Gene and protein expression were analyzed via qRT-PCR and western blot. Molecular interactions were confirmed through dual-luciferase reporter gene and RIP assays.

**Results:**

Hsa_circ_0004872, derived from exons 2 to 4 of the host gene MAPK1, demonstrated enhanced stability compared to its host MAPK1. Clinical data described that hsa_circ_0004872 was reduced in meningioma tissues and cell lines, and negatively correlated to poor survival rate of meningioma patients. Overexpression of hsa_circ_0004872 exhibited inhibitory effects on cell proliferation and promotion of apoptosis in vitro. Subsequent investigations unveiled a direct interaction between hsa_circ_0004872 and miR-190a-3p, leading to the activation of the PI3K/AKT signaling pathway through targeting PTEN. Notably, miR-190a-3p silence accelerated the apoptosis and proliferation inhibition of meningioma cells by inactivating PTEN/PI3K/AKT signaling, while miR-190a-3p overexpression showed an opposite effect, which greatly reversed the anti-tumor effects of hsa_circ_0004872 overexpression.

**Conclusion:**

In summary, our findings highlighted the intricate role of hsa_circ_0004872 in meningioma, shedding light on the regulatory mechanisms involving circ-RNAs in tumor progression. This positions hsa_circ_0004872 as a potential key regulatory factor in meningioma with implications for future therapeutic interventions.

**Supplementary Information:**

The online version contains supplementary material available at 10.1186/s12885-024-12084-1.

## Highlights

Hsa_circ_0004872 exhibits low expression in meningioma, correlating with a diminished survival rate among patients.

Overexpression of hsa_circ_0004872 impedes meningioma cell proliferation while enhancing apoptosis.

Hsa_circ_0004872 acts as a negative regulator of miR-190a-3p by functioning as a miRNA sponge.

Upregulation of miR-190a-3p counteracts the anti-tumor effects induced by hsa_circ_0004872 overexpression in vitro.

Inhibition of miR-190a-3p reduces cell proliferation and heightens apoptosis by targeting the PTEN/PI3K/AKT signaling pathway.

**Supplementary Information:**

The online version contains supplementary material available at 10.1186/s12885-024-12084-1.

## Introduction

Meningioma, constituting 36.6% of all primary central nervous system (CNS) malignancies [1], poses a significant health challenge. Classified into benign (Grade I), atypical (Grade II), and anaplastic (Grade III) tumors according to the 2021 WHO CNS tumor grading guidelines [2], malignant meningiomas (Grade II/III) exhibit high recurrence rates and dismal prognoses, profoundly impacting patient survival and quality of life [3]. Understanding the etiology of malignant meningioma is crucial for devising innovative treatment strategies.

It was well known that non-coding RNAs are the most of the transcription products of the human genome [4]. Circular RNAs (circRNAs) refer to a class of non-coding RNAs with closed loop structure [5]. It has been widely described that circRNAs are involved in the progression of human malignant tumors [6]. As proof, Chen et al. demonstrated that circRNA_0000285 expression was highly expressed in cervical cancer tissues, and its knockdown significantly suppressed cervical cancer cell growth and migration [7]. In addition, circ_102231 was significantly upregulated in gastric cancer tissues, and its silencing suppressed gastric cancer cell proliferation and invasion [8]. Hsa_circ_0004872 (circMAPK1), derived from exons 2 ∼ 4 of the mitogen-activated protein kinase 1 (MAPK1) gene [9]. Several studies have emphasized the function of hsa_circ_0004872 in tumor biology. For instance, hsa_circ_0004872 was found to be downregulated in oral squamous cell carcinoma, and its overexpression inhibited the proliferation, cell vitality and invasion of oral squamous cells [10]. However, the regulatory role of hsa_circ_0004872 in meningioma progression remains unexplored, necessitating further investigation.

CircRNAs, known for their role as competing endogenous RNAs (ceRNAs), function by sequestering microRNAs (miRNAs) and modulating their binding to target genes [11]. MiRNAs refer to non-coding RNAs with a length of about 22 nts, which are involved in various biological processes [12]. MiRNAs dysregulation is an important inducer for the pathogenesis and development of meningioma [13]. For instance, miRNA-145 was down-regulated in atypical and anaplastic meningiomas and could negatively regulate meningioma cell proliferation [14]. Recent focus has turned to miR-190a-3p in cancer research. Studies by Zhou et al. revealed elevated expression of miR-190a-3p in glioma tissues and cells. Its knockdown suppressed glioma cell proliferation while promoting apoptosis [15]. Conversely, in glioblastoma, miR-190a-3p facilitated tumorigenesis as a downstream target of LINC00657 [16]. These findings collectively underscore the cancer-promoting role of miR-190a-3p. However, the functions of miR-190a-3p in meningioma, as well as its regulatory relationship with hsa_circ_0004872, remain largely unexplored.

In the present study, we unraveled the inhibitory role of hsa_circ_0004872 in meningioma progression by negatively regulating miR-190a-3p expression, subsequently modulating the phosphatase and tensin homolog deleted on chromosome ten (PTEN)/phosphoinositide-3 kinase (PI3K)/protein kinase B (AKT) pathway. The findings contribute to the theoretical foundation for developing novel therapeutic strategies for meningioma.

## Materials and methods

### Cell culture

Human normal meningothelial cells (MECs, HUM-iCell-n002, iCell, Shanghai, China) and meningioma cells (IOMM-Lee, CRL-3370, ATCC, VA, USA), BEN‐MEN‐1 (GD-C00515294S, Sgdbio, Shanghai, China), CH157-MN (SR90102R, Xrshbio, Shanghai, China) and HBL-52 (GD-C00516386S, Sgdbio) were cultured in Dulbecco’s Modified Eagle’s medium (DMEM, Gibco, MD, USA) containing 10% fetal bovine serum (FBS, Gibco) at 37 °C with 5% CO_2_.

### RNA extraction and quantitative real-time polymerase chain reaction (qRT-PCR)

TRIzol reagent (Thermo Fisher Scientific, MA, USA) was used to extract total RNAs from meningeoma tissues and cell lines. Then, the cDNA was synthesized using the cDNA synthesis kit (Toyobo, Tokyo, Japan) and subsequently used for qRT-PCR assay using SYBR (Thermo Fisher Scientific). The relative expressions were normalized by GAPDH or U6 and calculated by the 2^−ΔΔCT^ method. The primers used in the study were listed as follows (5’-3’):

hsa_circ_0004872 (F): ACCTACTGCCAGAGAACCCT.

hsa_circ_0004872 (R): CAGGTTGGAAGGCTTGAGGT.

miR-190a-3p (F): CTAGGAGCTTCAGTAACGTTA.

miR-190a-3p (R): GAATTCGAATCGGTAACAGCT.

PTEN (F): CGACGGGAAGACAAGTTCAT.

PTEN (R): AGGTTTCCTCTGGTCCTGGT.

GAPDH (F): CCAGGTGGTCTCCTCTGA.

GAPDH (R): GCTGTAGCCAAATCGTTGT.

U6 (F): GTGCAGGGTCCGAGGT.

U6 (R): CTCGCTTCGGCAGCACA.

### Identification of hsa_circ_0004872

To confirm the specificity of the hsa_circ_0004872, the PCR products of hsa_circ_0004872 was amplified by divergent primer (F: 5‘-TGTTGAATTCCAAGCTCTGCTTA-3’, R 5’-TGTTGAATTCCAAGCTCTGCTTA-3’) or convergent primer (F: 5’-ACCTACTGCCAGAGAACCCT-3’, R 5’- CAGGTTGGAAGGCTTGAGGT-3’), and followed by separated on 1% agarose gel. The genomic DNA (gDNA) and cDNA were used as a PCR template. To further validate the stability of hsa_circ_0004872, IOMM-Lee and CH157-MN cells were exposed to RNase R (4U/mg, Epicentre Technologies, WI, USA) for 30 min at 37 ℃ or treated with 5 mg/ml actinomycin D (Sigma-Aldrich, MO, USA) for 0 h, 4 h, 8 h, 12 and 24 h. Afterwards, the total RNA was extracted as described above, and the abundance of hsa_circ_0004872 and liner MAPK1 gene were evaluated by qRT-PCR.

### Clinical sample collection

A total of 30 malignant meningioma tissues, along with paired adjacent normal tissues, were post-operatively collected from meningioma patients at Zhuzhou Hospital affiliated with Xiangya Medical College. Immediately following collection, the tumor and adjacent tissues were flash-frozen and stored in liquid nitrogen. The study protocol underwent rigorous review and approval by the Ethics Committee of Zhuzhou Hospital affiliated with Xiangya Medical College (Approval No. 2018K0345). All participants in the study provided informed consent.

### Cell transfection

Overexpression plasmids, including hsa_circ_0004872 (pcDNA3.1-circ), miR-190a-3p mimics/inhibitor, and their respective negative controls (pcDNA3.1), were procured from GenePharma (Shanghai, China). Transfection was carried out using Lipofectamine™ 3000 (Invitrogen, CA, USA) following the manufacturer’s instructions. Transfection efficiency was assessed using qRT-PCR.

### Western blot

Proteins were extracted using RIPA lysis buffer (Beyotime, Shanghai, China), and their concentrations were quantified with a BCA kit (Beyotime). Equal amounts of total proteins were separated by 12% SDS-PAGE and subsequently transferred onto a PVDF membrane (Millipore, Boston, MA, USA). The membranes were incubated overnight with specific antibodies, including B-cell lymphoma-2 (Bcl-2) (#26593-1-AP, Proteintech, Chicago, USA), Bax (#250599-2-Ig, Proteintech), Caspase3 (#19677-1-AP, Proteintech), PTEN (ab267787, Abcam, Cambridge, MA, USA), PI3K (#60225-1-Ig, Proteintech), p-PI3K (Abcam, ab278545), AKT (#10176-2-AP, Proteintech), p-AKT (#28731-1-AP, Proteintech), and GAPDH (#10494-1-AP, Proteintech). Subsequently, the blots were hybridized with secondary antibodies (Abcam, ab7090 or ab6728) for 60 min. Blot visualization was performed using a GEL imaging system (Bio-Rad, CA, USA), and the results were analyzed with ImageJ software.

### Subcellular fractionation

Nuclear and cytoplasmic RNA isolation was conducted using the PARISTM kit (Austin, TX, USA). Subsequently, RNA extraction followed the previously described method. Quantification of RNA samples was performed through qRT-PCR analysis, as outlined above. U6 served as a nuclear control, while GAPDH was utilized as a cytoplasmic control.

### Cell proliferation assay

Cell proliferation of meningeoma cells was measured by using a cell counting kit-8 (CCK-8) assay (CCK-8, Dojin, Japan). Briefly, IOMM-Lee and CH157-MN cells were cultured in 24-well plates (2 × 10^3^ cells/well) for 24 h and incubated with 10 µL of CCK-8 solution at 37 °C for 3 h. Absorbance was analyzed at 450 nm with a microplate spectrophotometer (Bioteke, Beijing, China).

### Cell apoptosis assay

Annexin V-FITC Apoptosis Detection Kit (Invitrogen) was used to detect apoptosis. After the corresponding treatments, IOMM-Lee and CH157-MN cells were harvested and washed with PBS solution. Then, cells were re-suspended in 500 µL of 1X Annexin-binding buffer and then incubated with 10 µL Annexin V-FITC and 5 µL PI stain for 10 min. Samples were immediately analyzed using flow cytometry (BD, NJ, USA).

### Dual-luciferase reporter gene assay

Hsa_circ_0004872/PTEN 3′-UTR fragments containing miR-190a-3p WT/MUT binding sites were amplified by PCR and inserted into pmirGLO reporter plasmids (Promega, WI, USA). Cells were co-transfected with circ_0004872-WT/PTEN-WT or circ_0004872-MUT/PTEN-MUT plasmids along with miR-190a-3p mimics or mimics NC using Lipofectamine™ 3000 (Invitrogen). Luciferase activity was assessed using a dual-luciferase reporter assay system (Promega).

### RNA immunoprecipitation (RIP) assay

The RIP assay was conducted using a RIP kit (Millipore). Briefly, cells were lysed with a complete RIP lysis buffer. Cell extracts were incubated with IgG (Abcam, ab172730) or Ago2 (Abcam, ab186733) antibodies at 4 °C overnight. Subsequently, the precipitated RNA was extracted as described above, and the expressions of hsa_circ_0004872 and miR-190a-3p were examined using qRT-PCR.

### In vivo experiments

IOMM-Lee cells were transfected with pcDNA3.1 or pcDNA3.1-circ_0004872, or co-transfected with pcDNA3.1-circ_0004872 and miR-190a-3p mimics. After transfection, cells were trypsinized, washed, and resuspended in PBS. Twenty male BALB/c nude mice (8-week-old, LABORATORY Animal Co, Ltd., Hunan, China) were randomly assigned to four groups: sham group, pcDNA3.1 group, pcDNA3.1-circ_0004872 group, and pcDNA3.1-circ_0004872 + miR-190a-3p mimics group (*n* = 5 per group). A 0.2 mL cell suspension containing 4 × 10^6^ cells was injected subcutaneously into the flank of each mouse. Tumor volumes were determined by measuring length (l) and width (w) and calculating volume (V) as follows: V = lw^2^/2. After 21 days, mice were euthanized, and tumor tissues were collected. The animal studies were approved by Zhuzhou Hospital affiliated to Xiangya Medical College (No.2018K0345).

### Statistical analysis

All data were obtained from three independent experiments. Statistical analysis was performed using SPSS 19.0 (IBM, Armonk, NY) and expressed as means ± SD. Between-group differences and multi-group comparisons were determined using Student’s t-test and one-way ANOVA, respectively. P values less than 0.05 were considered significant.

## Results

### Hsa_circ_0004872 was lowly expressed in meningioma and negatively correlated patient poor prognosis

**Fig. 1:**
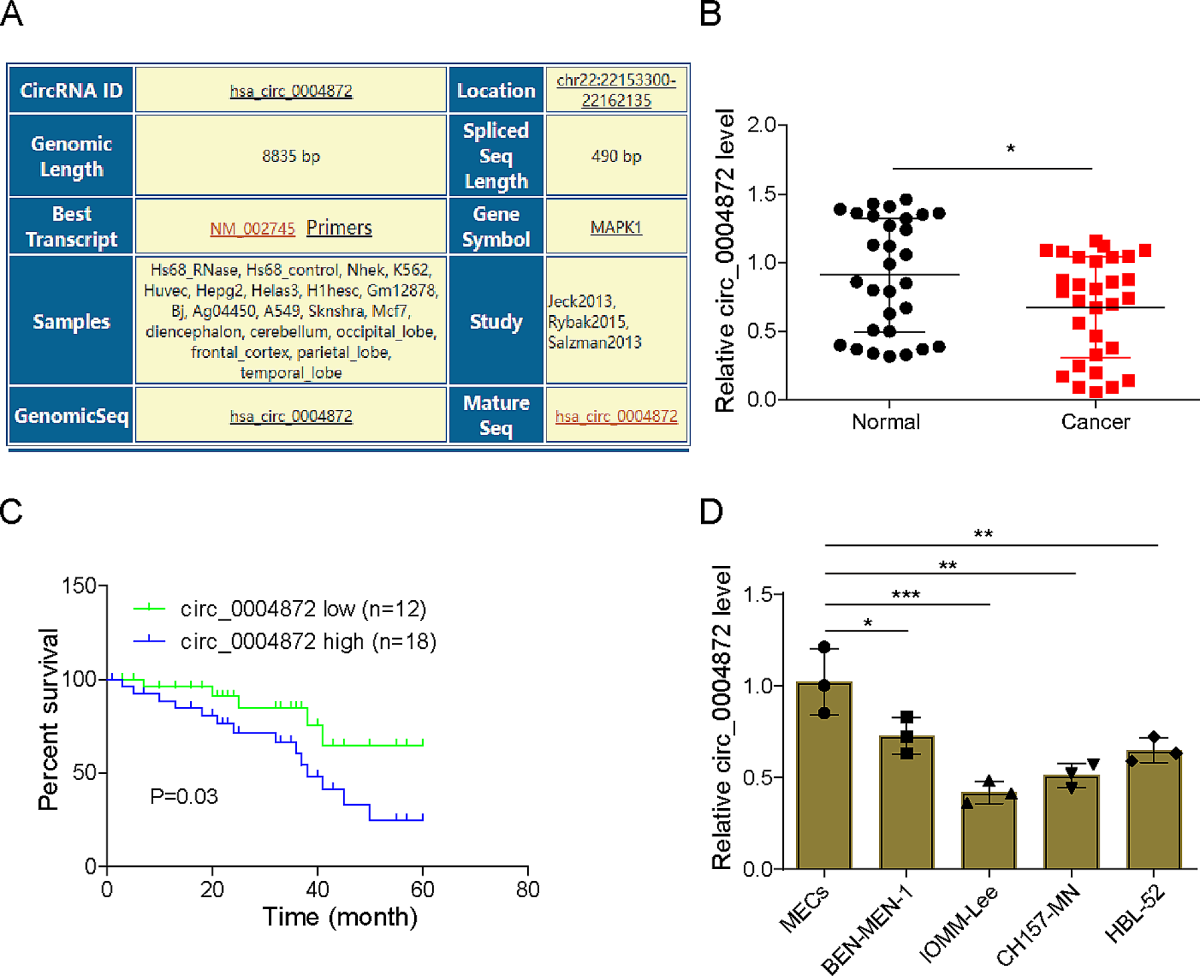
Hsa_circ_0004872 was lowly expressed in meningioma and negatively correlated patient poor prognosis. **(A)** The basic information about hsa_circ_0004872 obtained from the Circinteractome database was presented. **(B)** Expression levels of hsa_circ_0004872 in tumor and adjacent tissues from meningioma patients was detected qRT-PCR (*n* = 30). **(C)** Kaplan-Meier analysis showing the correlation between hsa_circ_0004872 expression and the survival rate of meningioma patients. **(D)** Detection of hsa_circ_0004872 expression in meningioma cells (IOMM-Lee, BEN‐MEN‐1, CH157-MN, and HBL-52) and human normal meningothelial cells (MECs) using qRT-PCR. Data are presented as mean ± SD from at least three independent experiments. *p < 0.05, **p < 0.01, ***p < 0.001

Hsa_circ_0004872 is a tumor suppressor gene in various human malignant tumors [10, 17], while the expression and the role of hsa_circ_0004872 in meningioma remain unknown. Hsa_circ_0004872 was generated by circularization of exons 2 to 4 of the MAPK1 gene according to circinteractome (https://circinteractome.nia.nih.gov/index.html) (Fig. 1A and S1A). To characterize the specificity of hsa_circ_0004872, we designed divergent and convergent primers to obtain the PCR amplification products, then the existence of hsa_circ_0004872 was further verified by 1% agarosegel electrophoresis. The results showed that hsa_circ_0004872 was amplified only from cDNA but not gDNA (Fig. S1B). Moreover, after digestion by Rnase R, it was observed that RNase R treatment could significantly reduce MAPK1 mRNA expression, but no significant change was observed in hsa_circ_0004872 expression (Fig. S1C). Similarly, after Actinomycin D treatment, the half-life of hsa_circ_0004872 was obviously longer than that of linear MAPK1 (Fig. S1D), revealing that hsa_circ_0004872 was more stable than linear MAPK1 mRNA. Subsequently, qRT-PCR showed that the level of hsa_circ_0004872 in human meningioma tissues was significantly higher than that in the normal group (Fig. 1B). Kaplan-Meier plotter analysis further showed that the high level of hsa_circ_0004872 was associated with good relapse free survival among meningioma patients (Fig. 1D).

### Hsa_circ_0004872 overexpression inhibited proliferation and promoted the apoptosis in vitro

**Fig. 2:**
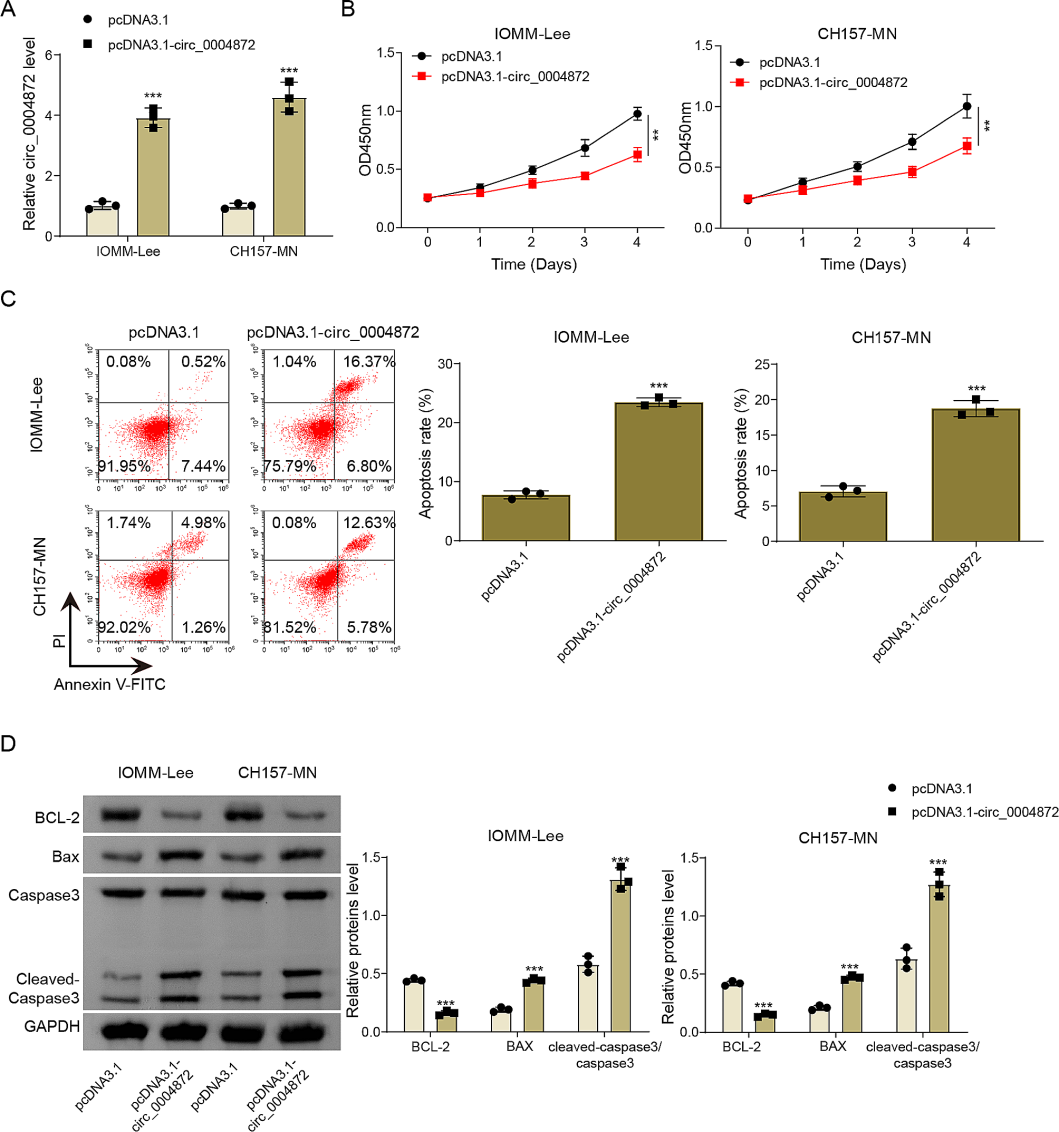
Hsa_circ_0004872 overexpression inhibited proliferation and promoted the apoptosis in vitro. IOMM-Lee and CH157-MN cells were transfected with either pcDNA3.1 or pcDNA3.1-circ_0004872. **(A)** hsa_circ_0004872 expression was evaluated by qRT-PCR. **(B)** Cell viability was assessed using the CCK8 assay. **(C)** Flow cytometry analysis determined cell apoptosis. **(D)** Protein levels of Bax, Bcl-2, Cleaved-caspase3, and Caspase3 were examined by Western blot. Data are presented as mean ± SD from at least three independent experiments. *p < 0.05, **p < 0.01, ***p < 0.001

To further study the potential function of hsa_circ_0004872 in the development of meningioma in vitro, hsa_circ_0004872 overexpression was induced in IOMM-Lee and CH157-MN cells. The transfection results were shown in Fig. 2A, hsa_circ_0004872 expression in IOMM‐Lee and CH157-MN cells was significantly increased by pcDNA3.1-circ_0004872 transfection. Additionally, transfection with pcDNA3.1- circ_0004872 notably elevated linear MAPK1 mRNA expression, but no significant changes were observed in MAPK protein levels (Fig. S2A-B). Subsequent CCK8 assay results demonstrated that hsa_circ_0004872 overexpression led to a significant inhibition of human meningioma cell proliferation (Fig. 2B). Flow cytometry analysis revealed that upregulation of hsa_circ_0004872 promoted human meningioma cell apoptosis (Fig. 2C). In line with these findings, hsa_circ_0004872 overexpression significantly increased the expressions of pro-apoptotic proteins (Bax and Cleaved-caspase 3) while inhibited the expression of the anti-apoptotic protein (Bcl2) (Fig. 2D). These results collectively underscored the tumor-suppressive role of hsa_circ_0004872 in the development of meningioma.

### Hsa_circ_0004872 directly targeted to miR-190a-3p

**Fig. 3:**
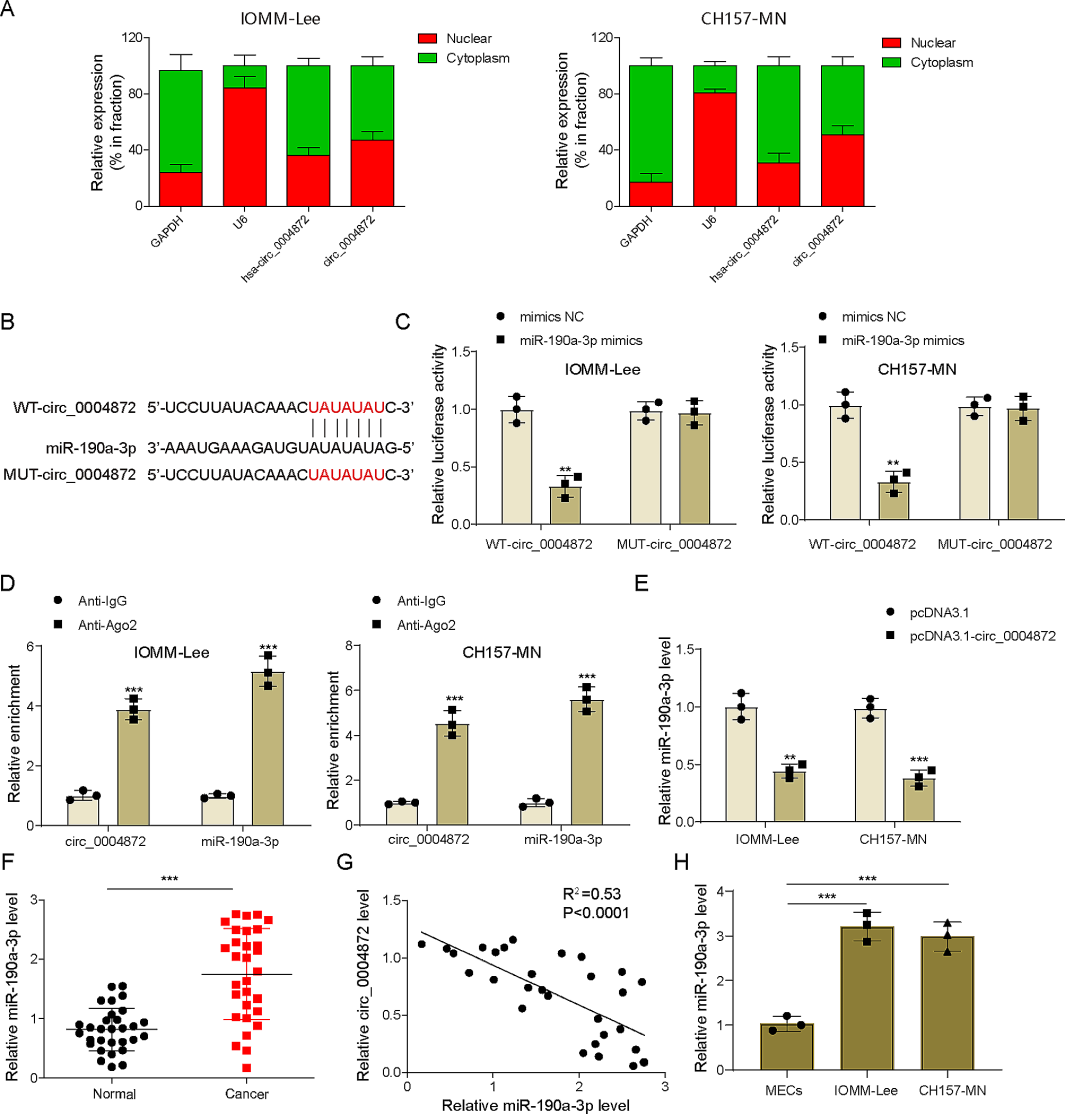
Hsa_circ_0004872 directly targets miR-190a-3p. **(A)** The subcellular localization of hsa_circ_0004872 was determined by qRT-PCR. (B) The potential binding site between hsa_circ_0004872 and miR-190a-3p. **(C-D)** The interaction between hsa_circ_0004872 and miR-190a-3p was assessed by dual-luciferase reporter gene and RIP assays. **(E)** MiR-190a-3p expression in IOMM-Lee and CH157-MN cells after pcDNA3.1 or pcDNA3.1-circ_0004872 transfection was measured by qRT-PCR. **(F)** MiR-190a-3p expression in tumor and adjacent tissues from meningioma patients was detected by qRT-PCR (*n* = 30). **(G)** Correlation analysis between hsa_circ_0004872 and miR-190a-3p expressions in clinical samples using Pearson’s correlation. **(H)** MiR-190a-3p expression in meningioma cells (IOMM‐Lee and CH157-MN) and human normal meningothelial cells (MECs) was determined by qRT-PCR. Data are presented as mean ± SD from at least three independent experiments. *p < 0.05, **p < 0.01, ***p < 0.001

As shown in Fig. 3A, the cytoplasmic localization of hsa_circ_0004872 in meningioma cells was prominently illustrated. Utilizing the Circinteractome database, the RNA sequence of hsa_circ_0004872 was acquired, revealing a discernible binding site between hsa_circ_0004872 and miR-190a-3p through sequence analysis and comparison (Fig. 3B). Subsequent findings from the dual-luciferase reporter gene assay underscored that miR-190a-3p mimics significantly reduced the luciferase activity presented by WT-circ_0004872, while exhibiting no significant effect on that of MUT-circ_0004872 (Fig. 3C). Meanwhile, RIP results displayed that hsa_circ_0004872 and miR-190a-3p were both enriched in the Ago2 antibody pull-down complex (Fig. 3D). Moreover, miR-190a-3p expression in IOMM-Lee and CH157-MN cells was notably diminished upon hsa_circ_0004872 overexpression (Fig. 3E). A parallel observation indicated an upregulation of miR-190a-3p in human meningioma tissues compared to the normal group (Fig. 3F). Notably, a negative correlation was discerned between miR-190a-3p and hsa_circ_0004872 expressions in clinical samples (Fig. 3G). Additionally, miR-190a-3p exhibited higher expression in IOMM‐Lee and CH157-MN cells relative to MECs (Fig. 3H). In summary, these findings collectively affirmed that hsa_circ_0004872 inhibited miR-190a-3p expression in meningioma by acting as a sponge for miR-190a-3p.

### miR-190a-3p negatively regulated the PTEN/PI3K/AKT signaling pathway

**Fig. 4:**
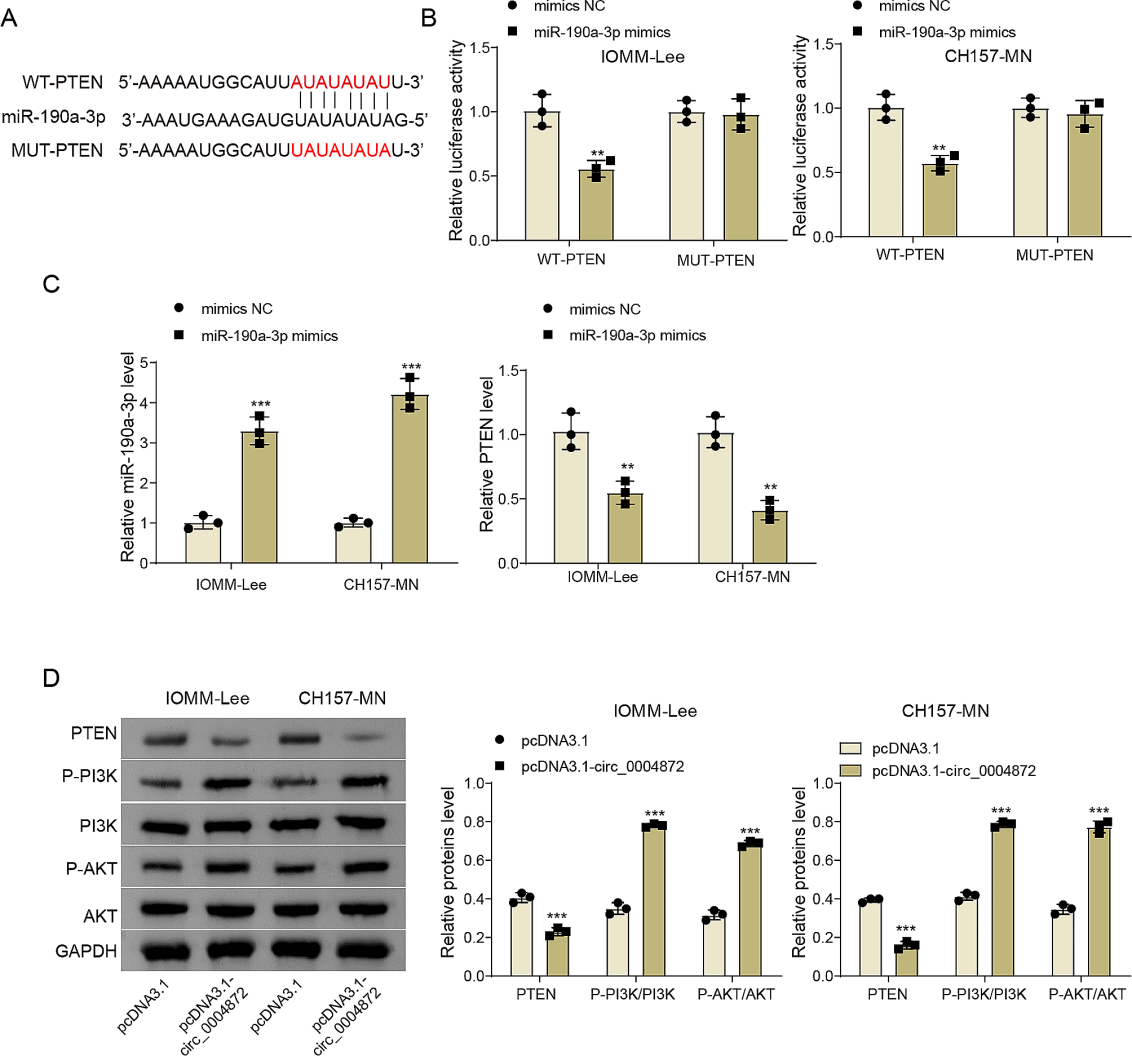
miR-190a-3p negatively regulated the PTEN/PI3K/AKT signaling pathway. **(A)** The potential binding site between PTEN and miR-190a-3p. **(B)** Dual-luciferase reporter gene assay showing the interaction between PTEN and miR-190a-3p. **(C)** MiR-190a-3p and PTEN expressions in IOMM-Lee and CH157-MN cells after mimics NC or miR-190a-3p mimics transfection were detected using qRT-PCR. **(D)** Western blot was employed to detect PTEN, PI3K, p-PI3K, AKT and p-AKT protein level in cells after mimics NC or miR-190a-3p mimics transfection. Data are presented as mean ± SD from at least three independent experiments. *p < 0.05, **p < 0.01, ***p < 0.001

PTEN/PI3K/AKT is an important pathway for regulating the signal transduction of various biological processes such as cell apoptosis, metabolism, cell proliferation and cell growth [18]. Herein, it was predicted that miR-190a-3p had a potential binding site to PTEN (Fig. 4A). Dual-luciferase reporter gene assay results demonstrated that miR-190a-3p overexpression significantly diminished the luciferase activity associated with WT-PTEN, while displaying no notable impact on MUT-PTEN (Fig. 4B). Additionally, qRT-PCR findings indicated that miR-190a-3p mimics transfection led to an elevation in miR-190a-3p expression and a concurrent reduction in PTEN expression in meningioma cells (Fig. 4C). Western blot assay also demonstrated that miR-190a-3p overexpression reduced PTEN protein level but increased p-PI3K and p-AKT levels in meningioma cells (Fig. 4D). Collectively, these findings underscored the functional interplay between miR-190a-3p and the PTEN/PI3K/AKT signaling pathway, positioning the latter as the downstream pathway influenced by miR-190a-3p in meningioma.

### MiR-190a-3p inhibition acted as a tumor-suppressing effect by modulating PTEN/PI3K/AKT pathway

**Fig. 5:**
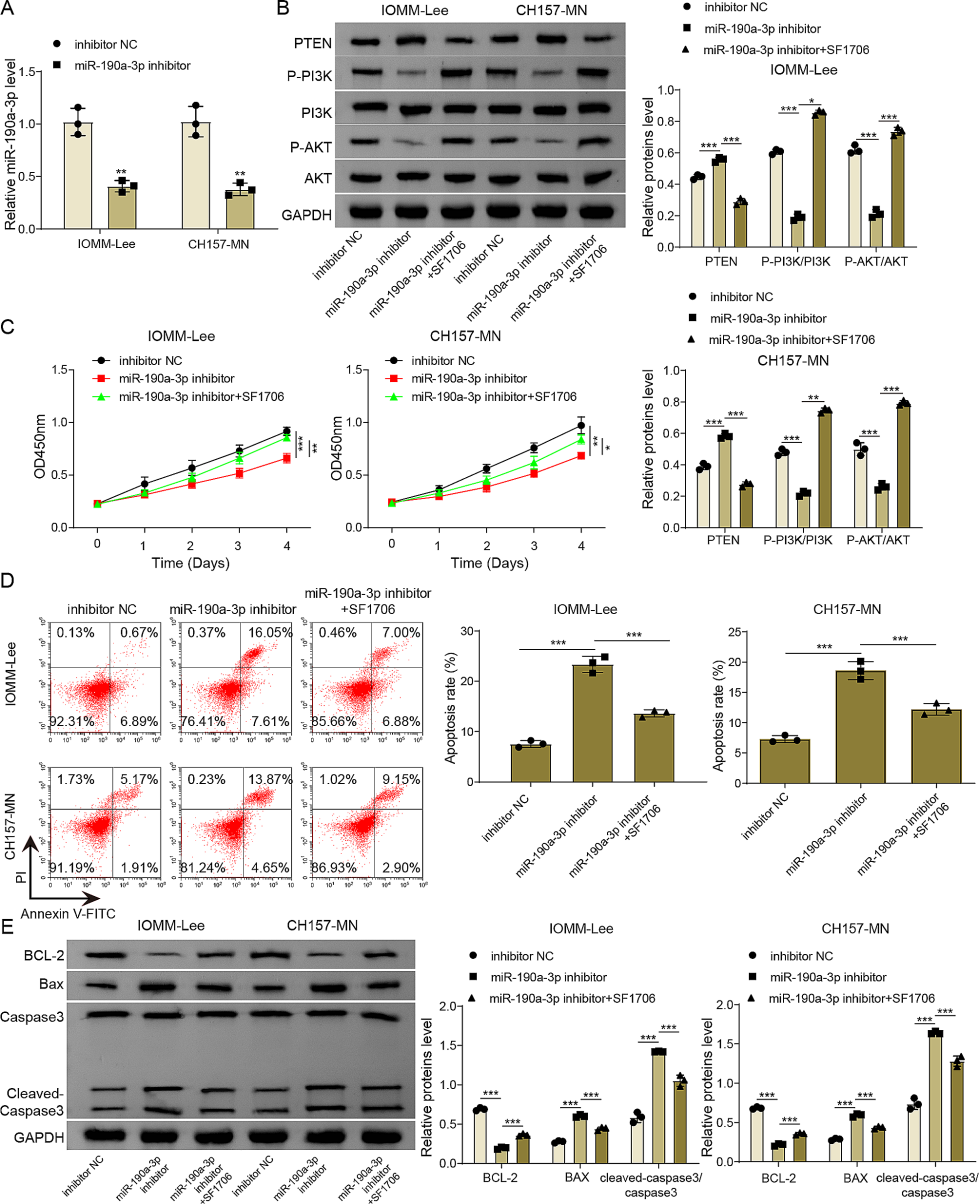
MiR-190a-3p inhibition acted as a tumor-suppressing effect by modulating PTEN/PI3K/AKT pathway. **(A)** IOMM-Lee and CH157-MN cells were transfected with inhibitor NC or miR-190a-3p inhibitor, and miR-190a-3p expression in cells was detected using qRT-PCR. IOMM‐Lee and CH157-MN cells were transfected with miR-190a-3p inhibitor or inhibitor NC combined with PTEN inhibitor (SF1670) treatment. **(B)** PTEN, PI3K, p-PI3K, AKT and p-AKT protein level were examined using western blot. **(C)** Cell viability was assessed using CCK8 assay. **(D)** Cell apoptosis was detected by flow cytometry. **(E)** Western blot was adopted to measure Bax, Bcl-2, Cleaved-caspase3, and Caspase3 protein levels. Data are presented as mean ± SD from at least three independent experiments. *p < 0.05, **p < 0.01, ***p < 0.001

To comprehensively investigate the impact of miR-190a-3p and PTEN/PI3K/AKT pathway in meningioma, we induced miR-190a-3p knockdown in meningioma cells and combined with PTEN inhibitor (SF1670) treatment. The transfection results were shown in Fig. 5A, miR-190a-3p inhibitor transfection significantly reduced miR-190a-3p expression in IOMM-Lee and CH157-MN cells. In addition, as shown in Fig. 5B, miR-190a-3p knockdown increased PTEN protein level and reduced p-PI3K and p-AKT levels in meningioma cells, while these effects were reversed by SF1670 treatment. Functional experiments subsequently revealed that miR-190a-3p knockdown restrained meningioma cell viability but promoted apoptosis. Importantly, these effects were abrogated by PTEN inhibition (Fig. 5C-D). Moreover, miR-190a-3p silencing significantly enhanced the protein levels of Bax and cleaved-caspase3 while concurrently inhibiting Bcl2 level (Fig. 5E). Significantly, these alterations were effectively counteracted following SF1670 treatment (Fig. 5E). To sum up, miR-190a-3p knockdown inhibited malignant behaviors of human meningioma cells by regulating the PTEN/PI3K/AKT pathway.

### miR-190a-3p overexpression partially eliminated the antitumor effect mediated by hsa_circ_0004872 overexpression

**Fig. 6:**
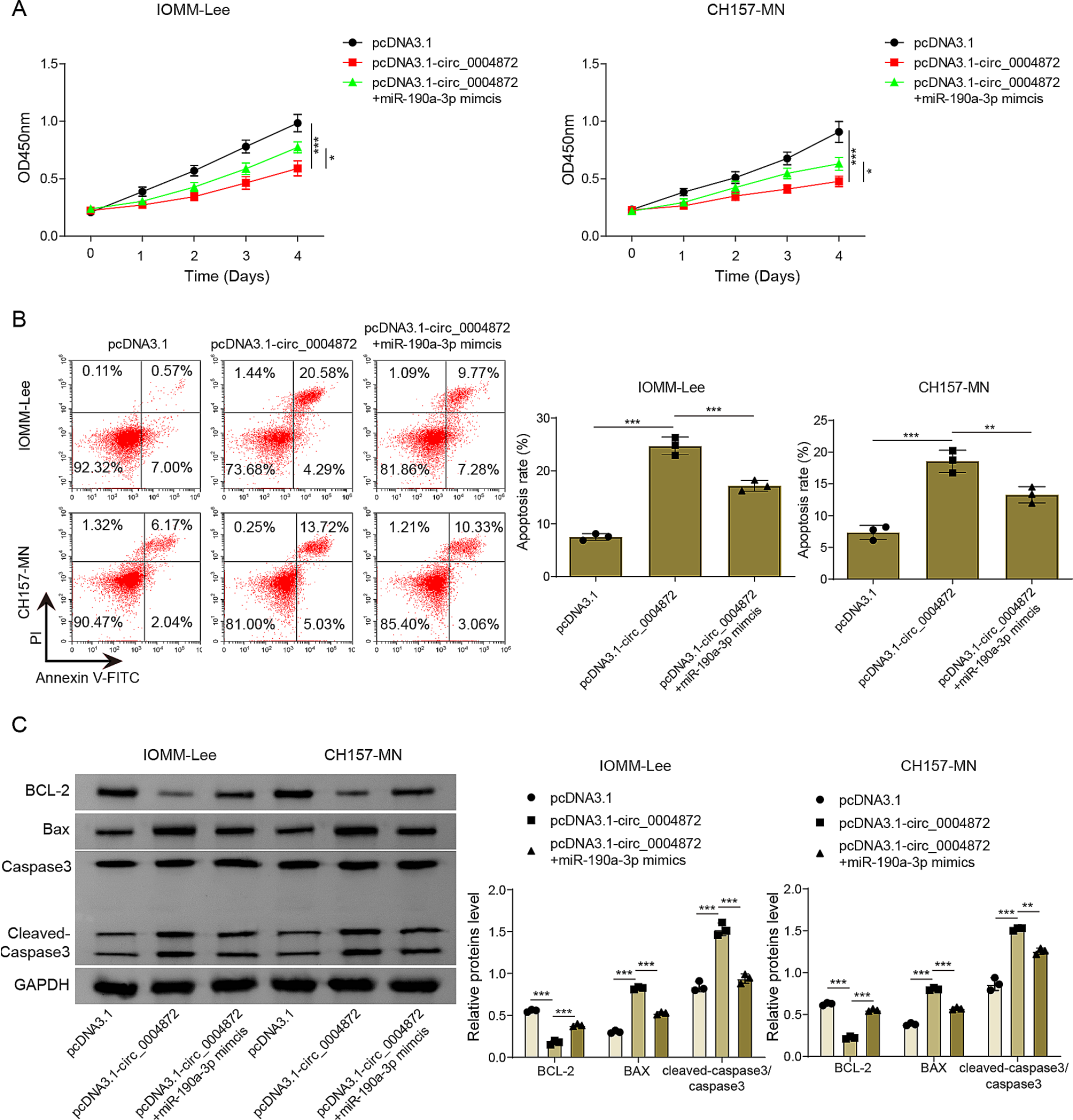
miR-190a-3p overexpression partially eliminated the antitumor effect mediated by hsa_circ_0004872 overexpression. IOMM-Lee and CH157-MN cells were transfected with pcDNA3.1, pcDNA3.1-circ_0004872 or co-transfected with pcDNA3.1-circ_0004872 plus miR-190a-3p mimics. **(A)** Cell viability was assessed using CCK8 assay. **(B)** Cell apoptosis was detected by flow cytometry. **(C)** Bax, Bcl-2, Cleaved-caspase3 and Caspase3 protein levels in cells were examined by Western blot. Data are presented as mean ± SD from at least three replicate experiments. *p < 0.05, **p < 0.01, ***p < 0.001

To unravel the intricate interplay between hsa_circ_0004872 and miR-190a-3p in orchestrating the development of human meningioma, both hsa_circ_0004872 and miR-190a-3p were co-overexpressed in human meningioma cells. It was observed that miR-190a-3p overexpression greatly reversed the inhibitory effect of hsa_circ_0004872 overexpression on meningioma cell viability (Fig. 6A). Additionally, the pro-apoptotic roles of hsa_circ_0004872 overexpression in meningioma cells were significantly abrogated upon miR-190a-3p overexpression (Fig. 6B). Further insights from western blot analysis elucidated that miR-190a-3p overexpression strikingly reverted the inhibitory impact of hsa_circ_0004872 upregulation on Bcl2 level in meningioma cells (Fig. 6C). Simultaneously, it negated the promotional effects of hsa_circ_0004872 upregulation on Bax and Cleaved-caspase 3 levels (Fig. 6C). In conclusion, hsa_circ_0004872 overexpression inhibited human meningioma cell proliferation and promoted the apoptosis by inhibiting miR-190a-3p expression.

### Hsa_circ_0004872 overexpression repressed tumor growth in vivo by inhibiting miR-190a-3p

**Fig. 7:**
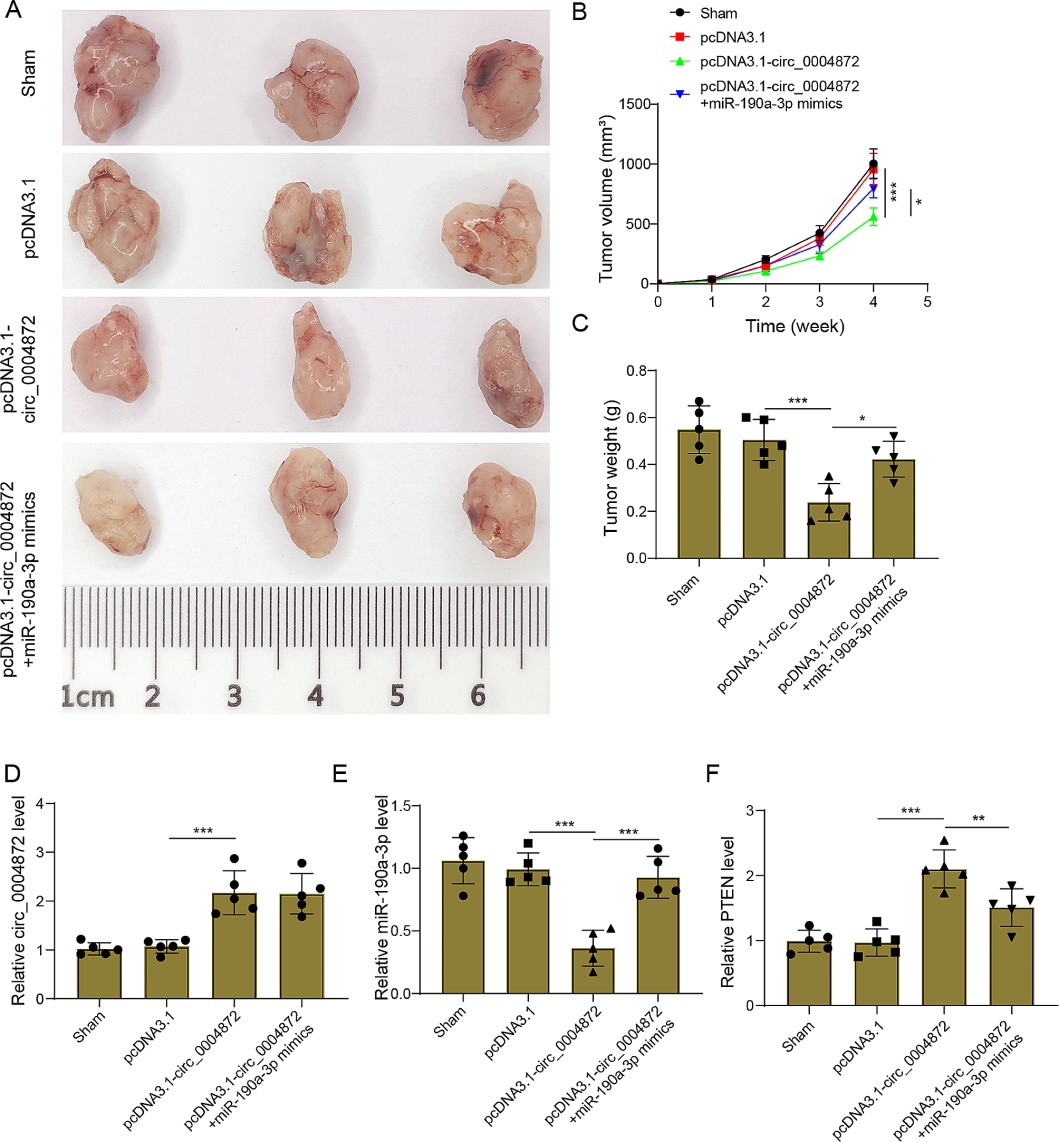
Hsa_circ_0004872 overexpression represses tumor growth in vivo by inhibiting miR-190a-3p. Nude mice were used to construct a Xenograft tumor model by subcutaneously injecting IOMM-Lee cells with different treatments (pcDNA3.1, pcDNA3.1-circ_0004872, or pcDNA3.1-circ_0004872 plus miR-190a-3p mimics). **(A-C)** Tumor tissues were collected, and images, tumor volume, and weight were measured. **(D-F)** The expressions of hsa_circ_0004872 **(D)**, miR-190a-3p **(E)**, and PTEN **(F)** in tumor tissues were examined by qRT-PCR. Data are presented as mean ± SD (*n* = 5). *p < 0.05, **p < 0.01, ***p < 0.001

Next, the function of has_circ_000482 in meningioma was further confirmed in vivo by constructing a Xenograft tumor model. As shown in Fig. 7A-C, in comparison to the pcDNA3.1 group, hsa_circ_0004872 overexpression exerted a profound inhibitory effect on tumor growth. Intriguingly, this anti-tumor impact of hsa_circ_0004872 upregulation was conspicuously reversed upon concurrent overexpression of miR-190a-3p (Fig. 7A-C). Furthermore, pcDNA3.1-circ_0004872 transfection significantly elevated the expressions of both hsa_circ_0004872 and PTEN, while concurrently diminishing miR-190a-3p expression (Fig. 7D-F). Conversely, co-transfection with miR-190a-3p mimics resulted in a marked increase in miR-190a-3p expression and a corresponding reduction in PTEN expression, albeit with no significant effect on hsa_circ_0004872 expression (Fig. 7D-F). In conclusion, hsa_circ_0004872 upregulation could suppress meningioma tumor growth in vivo by inhibiting miR-190a-3p.

## Discussion

Meningioma, the most prevalent intracranial tumor, exhibits a substantial incidence rate [19]. Despite the majority of meningiomas being categorized as benign, a concerning 90% of these tumors progress to malignancy [20]. While advances in meningioma treatment have transpired, the prognosis for malignant meningioma patients remains suboptimal [21]. Over the past decades, circRNAs have emerged as pivotal regulators in the development of various human malignancies. However, their specific role in the progression of meningiomas has remained elusive. This study unveiled a novel revelation, demonstrating that the upregulation of hsa_circ_0004872 served as a potent inhibitor of meningioma progression. This inhibitory effect was mediated through the intricate regulation of the miR-190a-3p/PTEN/PI3K/AKT pathway. The findings shed light on a previously undiscovered dimension of circRNA involvement in meningioma development.

Circular RNA (circRNA) stands out as a distinctive class of endogenous, single-stranded closed RNA molecules known for their stable structure, high conservation, and widespread expression across multiple species [22]. It has been widely revealed that circRNAs are involved in the initiation and development of human cancers by regulation of various biological processes, such as cell proliferation, invasion, and apoptosis [23]. As proof, circ_102958 was increased in GC tumor tissue, and its high level was positively correlated to the TNM stage and shorter survival [24]. In addition, it was previously illustrated that hsa_circ_001783 knockdown markedly inhibited breast cancer cell proliferation and invasion [25]. One notable circRNA, hsa_circ_0004872, is derived from the reverse splicing of MAPK1 exons 2, 3, and 4. MAPK1 plays an important role in regulating the proliferation, invasion, and migration of cancer cells [26]. Previous studies have demonstrated the antitumor effects of hsa_circ_0004872, including its inhibition of gastric cancer cell proliferation and invasion, ultimately leading to improved survival rates [27]. During our experiments, transfection with pcDNA3.1-circ_0004872 notably elevated linear MAPK1 mRNA expression, albeit without a corresponding change in MAPK protein level. This intriguing finding may be attributed to the partial base sequence overlap between hsa_circ_0004872 and linear MAPK1. Considering that has_circ_0004872 shares a part of the base sequence with linear MAPK1, but it is only a fragment, which does not contain the complete linear MAPK1 mRNA structure, such as translation initiation site. This may be the reason why hsa_circ_0004872 overexpression did not promote the expression of its MAPK1 protein. Notably, our data revealed elevated hsa_circ_0004872 level in meningioma tissues and cells, and its heightened expression correlated with favorable relapse-free survival among meningioma patients. Functional experiments demonstrated that hsa_circ_0004872 overexpression exerted inhibitory effects on human meningioma cell proliferation and tumor growth while promoting apoptosis. This groundbreaking study, to our knowledge, marked the first investigation into the functional role of hsa_circ_0004872 in meningioma. Furthermore, our findings suggested that the diminished expression of hsa_circ_0004872 may serve as a potential marker for poor prognosis in meningioma. This discovery hold promise for the development of innovative clinical diagnostic techniques, offering new avenues for improved prognostication in meningioma.

The intricate world of circRNAs encompasses a myriad of regulatory functions, including the modulation of transcription, splicing, stability, and translation of cytoplasmic mRNA. Operating as versatile players, circRNAs can interfere with signaling pathways through specific interactions with DNA, RNA, and proteins, relying on their subcellular localization [28]. A well-documented role of circRNAs involves acting as ‘sponges’ for miRNAs, engaging in competitive binding to neutralize the impact of miRNAs on mRNA translation [29]. Our investigation into the function of hsa_circ_0004872 in meningioma unveiled a compelling regulatory network, positioning hsa_circ_0004872 as a miRNA sponge, particularly for miR-190a-3p. Subsequent nuclear-cytoplasmic separation experiments affirmed the cytoplasmic localization of hsa_circ_0004872, reinforcing the hypothesis that its mode of action in meningioma involves miRNA sequestration. This pioneering study introduced a novel ceRNA regulatory network in meningioma, with hsa_circ_0004872 orchestrating its effects by sponging miR-190a-3p. MiR-190a-3p, the predominant mature form of miR-190, assumed a crucial role in various diseases, particularly in the context of malignant tumors [30]. The role of miR-190a-3p in different cancers can be dual, either promoting or suppressing tumorigenic processes. For example, miR-190a-3p exhibited elevated levels in glioma, where it stimulated proliferation and migration by targeting YOD1 [15]. Moreover, miR-190a-3p was decreased in cervical cancer, and play as a suppressor to repress invasion by targeting KLF6 [31]. Despite the wealth of knowledge on miR-190a-3p in diverse malignancies, its function in meningioma remains elusive. Our study bridged this knowledge gap by revealing that miR-190a-3p was markedly upregulated in meningioma tissues, and its expression was negatively correlated with hsa_circ_0004872 expression. Our results subsequently revealed that hsa_circ_0004872 operated as the sponge of miR-190a-3p to negatively regulate miR-190a-3p expression in meningioma cells. In addition, as expected, miR-190a-3p overexpression reversed the inhibitory effect of hsa_circ_0004872 overexpression on meningioma cell malignant behaviors. Collectively, these findings underscored the pivotal role of the hsa_circ_0004872/miR-190a-3p regulatory axis in steering the progression of malignant meningioma. This axis shed light on the intricate molecular mechanisms at play but also holds promise as a potential diagnostic and prognostic marker for meningioma.

PTEN, a dual-function protein and lipid phosphatase, stands as a formidable tumor suppressor, wielding a pivotal role in constraining tumor cell proliferation [32]. Its inactivation has been entwined with the genesis and progression of diverse human malignancies, spanning from colon cancer to lung cancer [33, 34]. The crux of PTEN’s functionality lies in its ability to negate the effects of PI3K, with its primary substrate being PIP3. The dysregulation of PTEN unleashes a cascade of events, notably the hyperactivation of the PI3K/AKT signaling pathway, a phenomenon associated with the accelerated progression of malignant tumors, including meningioma [35, 36]. Our investigation delved into the intricate dynamics of the miR-190a-3p/PTEN relationship in meningioma cells, revealing a direct negative regulation exerted by miR-190a-3p on PTEN expression. This interaction extended its impact to the downstream effectors of the PTEN/PI3K/AKT pathway, with miR-190a-3p overexpression elevating p-PI3K and p-AKT levels in meningioma cells. Importantly, the consequential inhibitory effects on malignant behaviors of meningioma cells due to miR-190a-3p knockdown were effectively reversed by PTEN inhibition. The broader context of this regulatory network encompasses the hsa_circ_0004872/miR-190a-3p/PTEN axis, where hsa_circ_0004872 acted as an orchestrator, influencing the expression and function of miR-190a-3p, which in turn governed the PTEN/PI3K/AKT signaling pathway. These findings provided a nuanced understanding of the intricate functions and potential mechanisms underlying the hsa_circ_0004872/miR-190a-3p/PTEN network in meningioma. This comprehensive elucidation of the ceRNA mechanisms at play not only enhanced our understanding of the molecular underpinnings of meningioma but also positioned this regulatory axis as a potential source of novel biomarkers for prognostic evaluation in meningioma patients.

In summary, this pioneering study shed light on the previously unexplored role of hsa_circ_0004872 in thwarting the progression of malignant meningioma. Through its intricate regulatory dance with the miR-190a-3p/PTEN/PI3K/AKT axis, hsa_circ_0004872 emerged as a potent suppressor of malignant behaviors in meningioma cells. These discoveries deepen our comprehension of the molecular intricacies underpinning meningioma, and further provides a solid theoretical foundation for the development of innovative approaches in the battle against malignant meningioma.

### Electronic supplementary material

Below is the link to the electronic supplementary material.

Supplementary Material 1

Supplementary Material 2

Supplementary Material 3

Supplementary Material 4

Supplementary Material 5

## Data Availability

The data underlying this article will be shared on reasonable request to the corresponding author.

## Abbreviations

CNS: Central nervous system
circRNAs: Circular RNAs
MAPK1: Mitogen-activated protein kinase 1
ceRNAs: Competing endogenous RNAs
miRNAs: MicroRNAs
PTEN: Phosphatase and tensin homolog deleted on chromosome ten
PI3K: Phosphoinositide-3 kinase
AKT: Protein kinase B
CCK-8: Cell counting kit-8
RIP: RNA Immunoprecipitation
qRT-PCR: Quantitative real-time polymerase chain reaction
