# Supplementary material for: Hsa_circ_0004872 alleviates meningioma progression by sponging miR-190a-3p/PTEN signaling
Source: BMC Cancer. 2024 Mar 18;24:345. doi: 10.1186/s12885-024-12084-1 (PMC10949562; doi:10.1186/s12885-024-12084-1)
Supplement: Supplementary file 1 — Supplementary Material 1 [file 12885_2024_12084_MOESM1_ESM.docx]

**Figure S1. The identification of hsa_circ_0004872 in human meningioma.**

(A) The detailed information of hsa_circ_0004872 was presented. (B) The existence of hsa_circ_0004872 was detected by using agarose gel electrophoresis. (C) The abundance of linear MAPK1 mRNA and hsa_circ_0004872 were analyzed by qRT-PCR after RNase R treatment. (D) The levels of linear MAPK1 mRNA and hsa_circ_0004872 were detected by qRT-PCR in cells after Actinomycin D treatment. The measurement data were presented as mean ± SD. All data was obtained from at least three replicate experiments. **p* < 0.05, ***p* < 0.01, ****p* < 0.001.

**Figure S2. The regulatory effects of circ_0004872 overexpression on MAPK1 expression**

IOMM‐Lee and CH157-MN cells were transfected pcDNA3.1 or pcDNA3.1-circ_0004872, and the mRNA (A) and protein (B) levels of MAPK1 in cells were determined by qRT-PCR and western blot, respectively. The measurement data were presented as mean ± SD. All data was obtained from at least three replicate experiments. **p* < 0.05, ***p* < 0.01, ****p* < 0.001.
