# Supplementary figures and images for: Hsa_circ_0004872 alleviates meningioma progression by sponging miR-190a-3p/PTEN signaling
Source: BMC Cancer. 2024 Mar 18;24:345. doi: 10.1186/s12885-024-12084-1 (PMC10949562; doi:10.1186/s12885-024-12084-1)

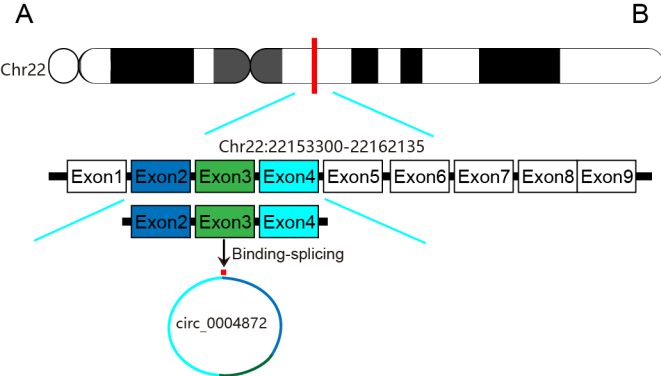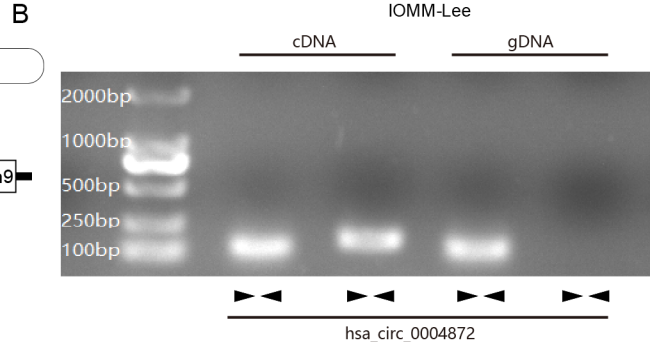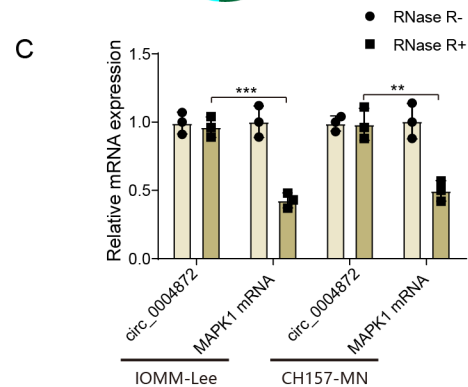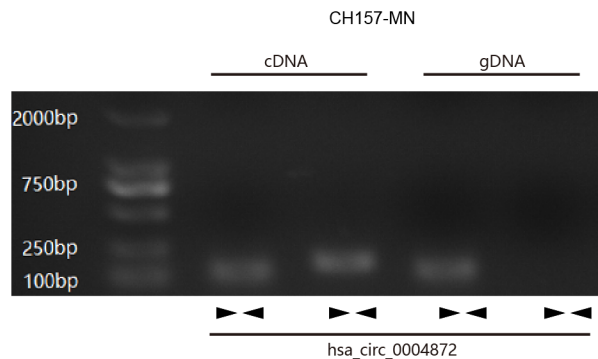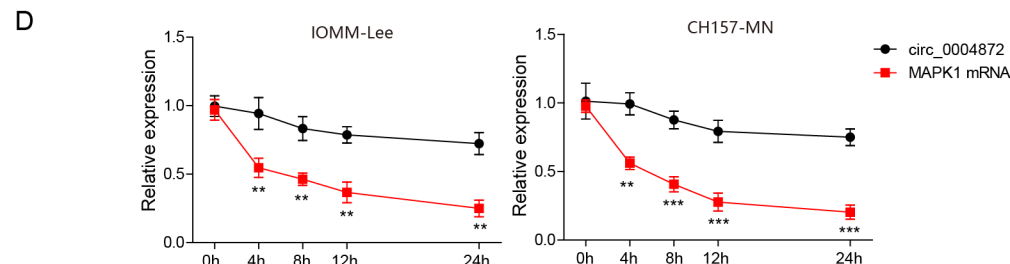

Supplement: Supplementary file 2 — Supplementary Material 2 [file 12885_2024_12084_MOESM2_ESM.pdf]

A

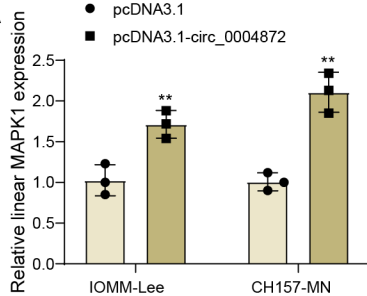

B

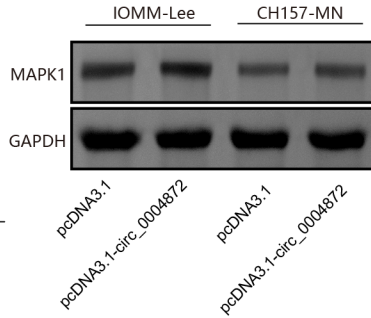

Relative Protein level of MAPK1

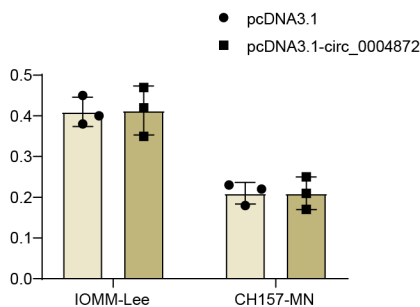

Supplement: Supplementary file 3 — Supplementary Material 3 [file 12885_2024_12084_MOESM3_ESM.pdf]

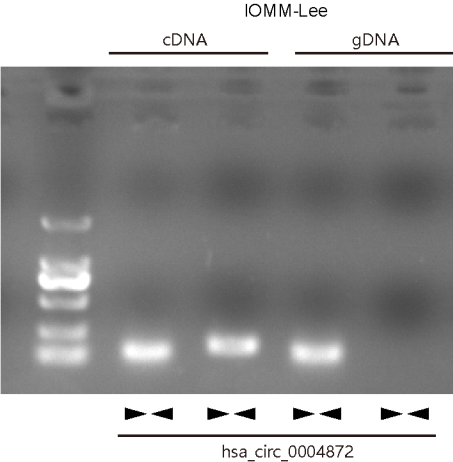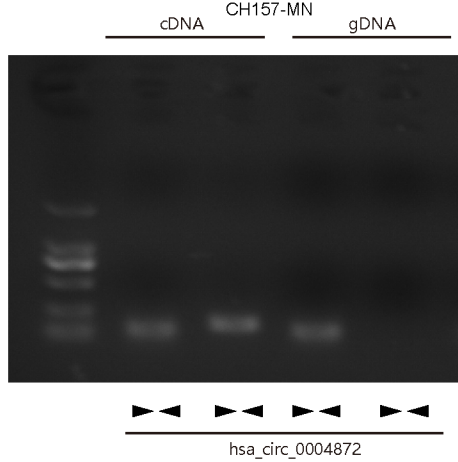

Supplement: Supplementary file 4 — Supplementary Material 4 [file 12885_2024_12084_MOESM4_ESM.pdf]
