# Supplementary material for: Hsa_circ_0004872 alleviates meningioma progression by sponging miR-190a-3p/PTEN signaling
Source: BMC Cancer. 2024 Mar 18;24:345. doi: 10.1186/s12885-024-12084-1 (PMC10949562; doi:10.1186/s12885-024-12084-1)
Supplement: Supplementary file 5 — Supplementary Material 5 [file 12885_2024_12084_MOESM5_ESM.pdf]

2D

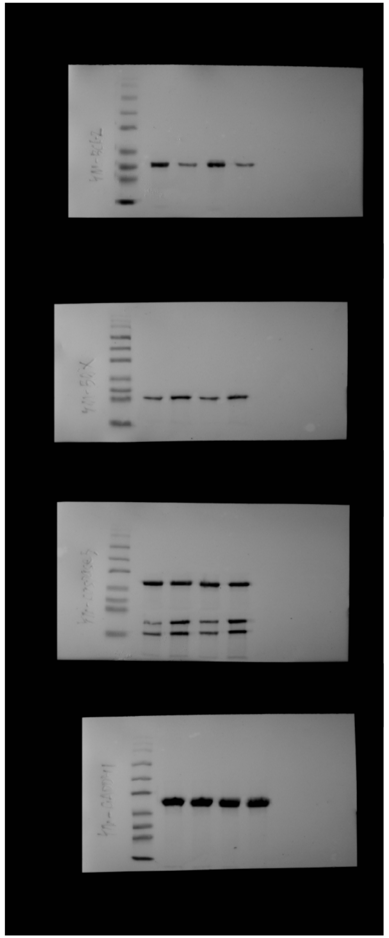
BCl-2
Bax
Caspase3
GAPDH

4D

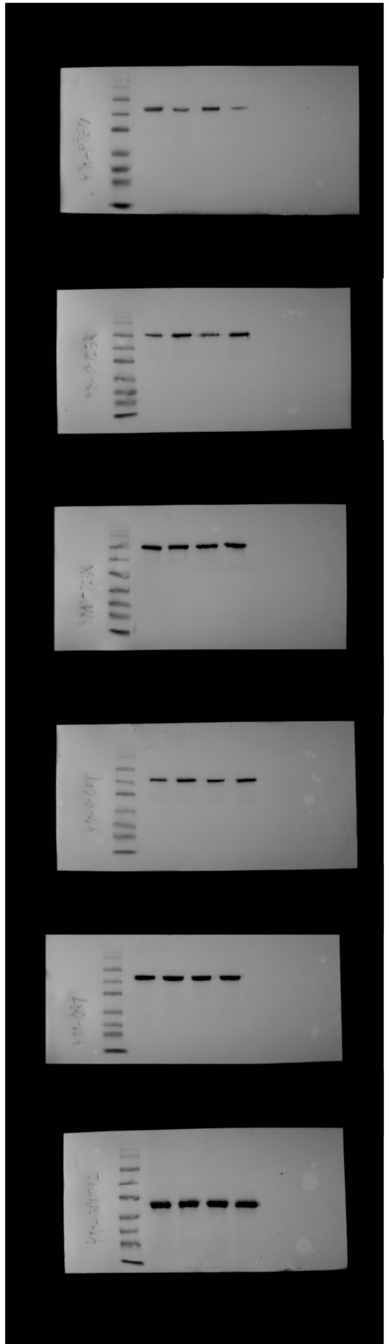
PTEN
p-PI3K
PI3K
p-AKT
AKT
GAPDH

5B

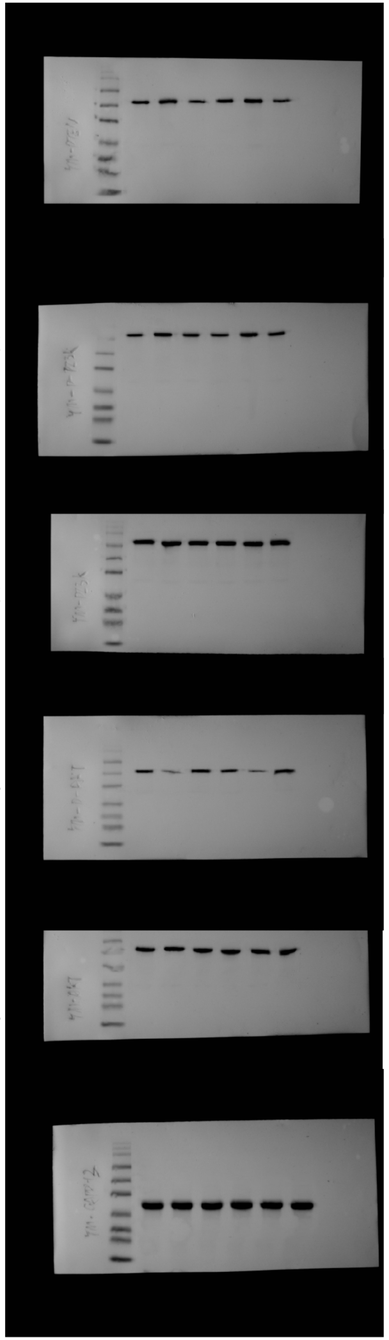
PTEN
p-PI3K
PI3K
p-AKT
AKT
GAPDH

5E

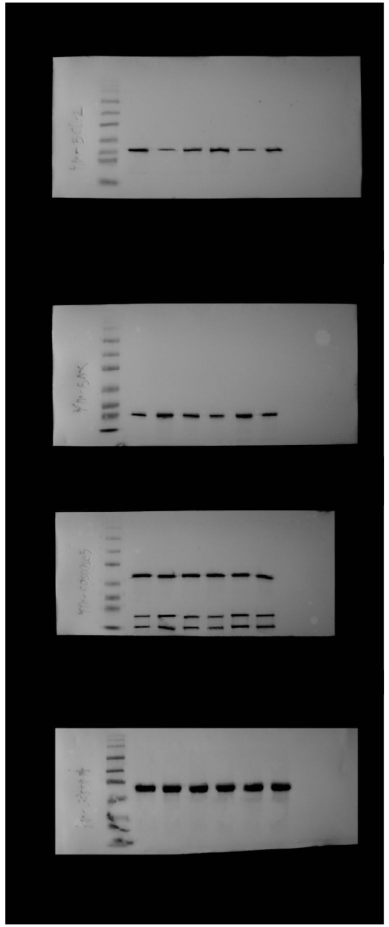
BCl-2
Bax
Caspase3
GAPDH

6C

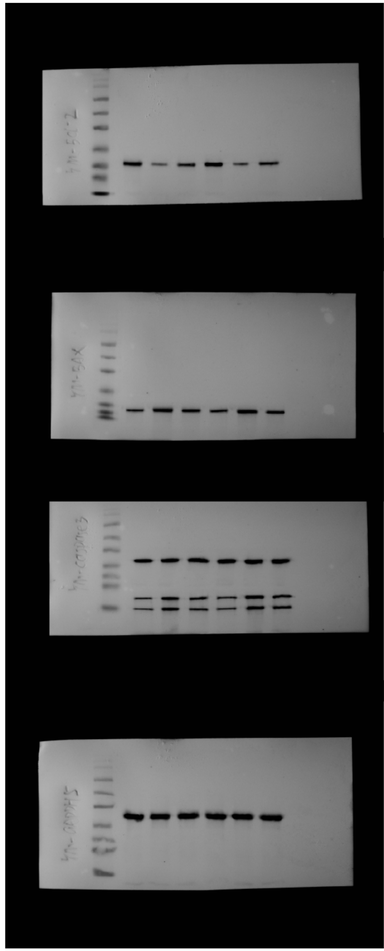
BCl-2
Bax
Caspase3
GAPDH
